# 

**Published:** 1887-01

**Authors:** 


### OUR ADVERTISERS.

LIEBIG PHARMACAL CO., NEW YORK.—See advertisement of this company to be found on page 26, and write them for samples of their goods.

GEORGE MUSE, ATLANTA.—This gentleman has the largest clothing store in Georgia and is a first-class man in every respect. Parties dealing with him will find him perfectly reliable. He promises to every one entire satisfaction. See his ad.

STEPHENS' SADDLE BAGS.—Observe the charge in the advertisement of Geo. K. Hopkins & Co., manufacturers of these justly celebrated bags. They are superior to any bag we know.

CODMAN & SHURTLEFF, BOSTON.—This well-known house has an advertisement elsewhere in THE JOURNAL, to which we invite the attention of our readers. We shall have more to say of them in another issue. See their advertisement, and write them for any goods you may need in their line.

RECTAL ALIMENTATION.—Peptonized milk, as manufactured by Messrs. Fairchild Bros. & Foster, of New York, is said to be the ideal substance for nutritive enema. It does not irritate the bowel, is perfectly absorbable and may be used almost indefinitely without causing any local disturbance. If you have not tried it, send for samples and give it a trial. You will be pleased with it.

SUCCUS ALTERANS (MCDADE).—The late Dr. J. Marion Sims, and many other physicians of note, both in this country and Europe, have given this remedy their unqualified endorsement in the treatment of syphilis. It is manufactured by Messrs. Eli Lilly & Co., of Indianapolis, one of the best-known houses in this country. See the advertisement next to "Correspondence," and if you would know more of the remedy address the firm for the *Monographia Syphilitica* for January, 1887. It is the latest publication of Dr. McDade, the gentleman who first introduced this remedy to the profession.

EISNER & MENDELSON CO., PHILADELPHIA.—These gentlemen have an attractive advertisement in this issue next to Book Reviews, to which we direct the attention of our readers. They are the sole agents for Johann Hoff's Extract of Malt, a preparation which bears the endorsement of prominent physicians everywhere. Read the advertisement and observe instructions closely, that you may always get the genuine article when you order malt extract.

PARKE, DAVIS & CO., DETROIT.—We desire to direct attention to the new advertisement of this house to be found next to clinic reports in this issue of THE JOURNAL. No house in the country makes superior pharmaceutical preparations to this one. Their name is a guarantee that the preparations manufactured by them are what is claimed for them. See their advertisement and write them for samples of any goods you may desire to test.

PEACOCK'S BROMIDES.—W. H. Wolford, M. D., Chicago, Ill., says: I have used Peacock's bromides in a number of cases with the best results, especially in epilepsy, one case in particular, C. S., a railroad man, having been compelled to quit work on account of the paroxysms coming on every day. After one week's treatment with Peacock's bromides, the attacks were considerably lessened; now, after two months' treatment, he seems entirely cured and has resumed work. Any case where there is a nerve sedative indicated I can cheerfully recommend Peacock's bromides.

